# Pretarsal Augmentation of the Lower Eyelids Using Fat Grafts in Asian Patients

**DOI:** 10.1093/asjof/ojad108

**Published:** 2023-11-29

**Authors:** Tsutomu Mizuno, Atsushi Yamamoto

## Abstract

**Background:**

Surgical procedures are required to achieve long-term pretarsal roll fullness of the lower eyelids with a satisfactory appearance.

**Objectives:**

To assess the efficacy of a surgical method using fat grafts for pretarsal augmentation of the lower eyelids.

**Methods:**

We conducted a retrospective clinical study that included 12 Japanese patients (24 lower eyelids) who underwent pretarsal augmentation of the lower eyelids using fat grafts and were followed up for more than 6 months. Morphometric measurements of the projection, width, and area of the pretarsal roll fullness of the lower eyelids were performed. Additionally, surgical outcomes and complications were assessed.

**Results:**

Pretarsal roll fullness of the lower eyelids was achieved for more than 6 months postoperatively. The surgery resulted in enlarged eyes with sufficient pretarsal roll fullness, and all patients were satisfied with the aesthetic outcomes. The mean projection (1.6 ± 1.0 vs 0.5 ± 0.6 mm), width (7.4 ± 1.5 vs 4.4 ± 3.6 mm), and area (143.5 ± 42.2 vs 32.8 ± 39.6 mm^2^) of the pretarsal roll fullness of the lower eyelids were significantly greater postoperatively than preoperatively (*P* < .05). No early postoperative complications were observed during the 6- to 12-month follow-up period. However, overcorrections were observed in 2 cases (16.7%) due to late-onset fat overgrowth, which was corrected by revision surgery.

**Conclusions:**

Fat grafting can help achieve sufficient pretarsal roll fullness of the lower eyelids. The technique presented herein is a simple and reliable alternative surgical procedure for creating pretarsal roll fullness of the lower eyelids.

**Level of Evidence: 4:**

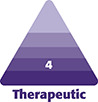

Cultural acceptance of pretarsal roll fullness differs between Asians and Caucasians.^1^ Among Asians, pretarsal roll fullness of the lower eyelids has recently gained popularity as it produces a more attractive and youthful appearance.^2^ Hyaluronic acid filler injection is a simple nonsurgical method used to achieve pretarsal roll fullness of the lower eyelids and has become a popular aesthetic procedure in East Asia.^3^ However, the effect of the injected hyaluronic acid filler is only temporary, as it is gradually absorbed by the body. Therefore, an effective surgical procedure with long-term benefits is required; however, reports regarding surgical achievement of pretarsal roll fullness of the lower eyelids are limited. This study describes a surgical procedure using fat grafts for pretarsal augmentation of the lower eyelid.

## METHODS

### Patients

This retrospective study included 22 consecutive Japanese patients who underwent pretarsal augmentation of the lower eyelids using fat grafts between May 2013 and September 2022. Ten patients who underwent lower eyelid blepharoplasty or were followed up for <6 months were excluded; finally, 12 patients (24 lower eyelids) were included. The clinical outcomes and complications were assessed by evaluating the preoperative and postoperative digital photographs and medical records of the patients. At 6 to 12 months postsurgery, all patients were asked to rate their satisfaction with their aesthetic outcome, on a scale of satisfied, fair, or poor. The survey was conducted by a blinded evaluator. All patients provided informed consent; however, Institutional Review Board approval could not be obtained because the study was conducted at the authors' private clinic. All procedures performed in this study, involving human participants, were in accordance with the ethical standards of the institutional and/or national research committee and with the 1964 Declaration of Helsinki and its later amendments or comparable ethical standards.

### Surgical Technique

Local anesthesia was administered using 1% xylocaine and epinephrine (1:100,000). Approximately, 2 mL of fat tissue without dermis was harvested from the inside of the upper arm, which was cut into small pieces (4-6 mm in size) and kept hydrated with a saline solution until use.

**Figure 1.**
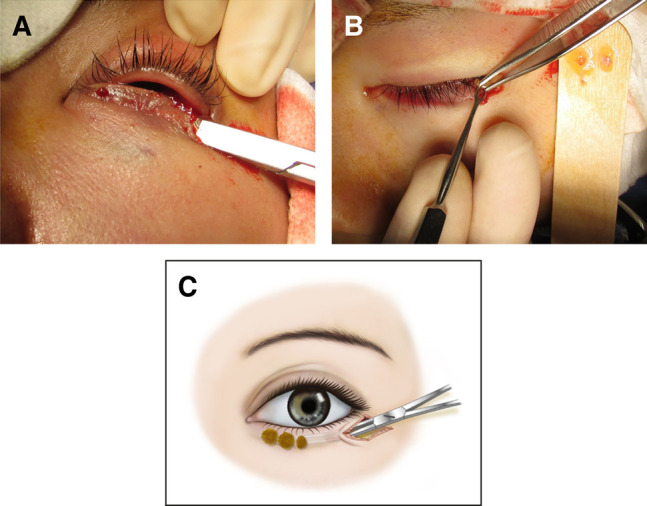
Surgical technique for pretarsal augmentation of the lower eyelids using fat grafts for a 27-year-old male patient. (A) After a small skin incision is made at the lateral canthus along the subciliary crease, the skin blunt undermining is performed using delicate straight scissors to create a subcutaneous tunnel in the pretarsal region of the lower eyelid. (B) Fat grafts are introduced from a lateral canthus skin incision using mosquito or Adson forceps. (C) An illustration of the surgical technique. Fat grafts are placed in the subcutaneous tunnel, which is created in the lower eyelid.

A small skin incision was made at the lateral canthus along the subciliary crease of the lower eyelid. Skin blunt undermining was performed using delicate straight scissors to create a subcutaneous tunnel in the pretarsal region of the lower eyelid (Figure 1A). The eyelash line was used as the upper border of the subcutaneous dissection. The desired size of the pretarsal roll fullness was determined according to the lower border of the dissected subcutaneous tunnel per surgeon discretion. Fat grafts were introduced through a skin incision at the lateral canthus using Adson or mosquito forceps (Figure 1B, C) and subsequently placed in the subcutaneous tunnel of the lower eyelid. A surgical probe was used to distribute the fat grafts medially into the distant dissected space. For each eyelid, 0.2 to 0.6 mL of fat graft was used. The skin incisions were closed using 7-0 nylon sutures, which were removed 1 week after the operation. The surgical procedure for the pretarsal augmentation of the lower eyelids using fat grafts is demonstrated in Video 1, available online at www.aestheticsurgeryjournal.com.

### Morphometric Measurements

The surgical outcomes were evaluated by comparing the morphometric parameters measured using facial photographs. Each patient held a ruler close to their face while a photograph was taken. Digital photographs were objectively assessed using ImageJ software (National Institutes of Health, Bethesda, MD).

**Figure 2.**
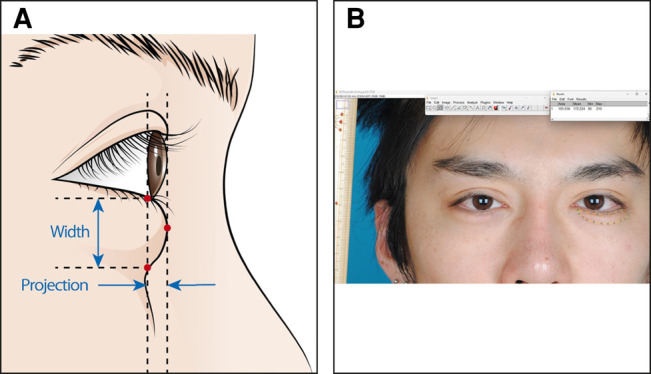
Morphometric parameters. (A) The projection and width of the pretarsal roll fullness of the lower eyelid are measured from the photographic profile view. (B) The area (yellow circle) of the pretarsal roll fullness of the lower eyelid is measured from the photographic frontal view of a 27-year-old male patient.

The projection and width of the pretarsal roll fullness of the lower eyelids were measured using a photographic profile view. The parameters of the lower eyelids are presented in Figure 2A. The projection of the pretarsal roll fullness was defined as the distance between the top of the pretarsal roll fullness and a line connecting the upper and lower edges of the pretarsal roll fullness of the lower eyelids. The width of the pretarsal roll fullness of the lower eyelids was defined as the distance between the upper and the lower edges. The area of the pretarsal roll fullness of the lower eyelids was measured using a photographic frontal view (Figure 2B).

### Statistical Analysis

The mean and standard deviation of each variable was calculated. Differences between preoperative and postoperative morphometric measurements were analyzed using the Wilcoxon signed-rank test. Differences were considered statistically significant at *P* < .05. Data were analyzed using IBM SPSS statistics, version 29 (IBM Corp., Armonk, NY).

## RESULTS

**Figure 3.**
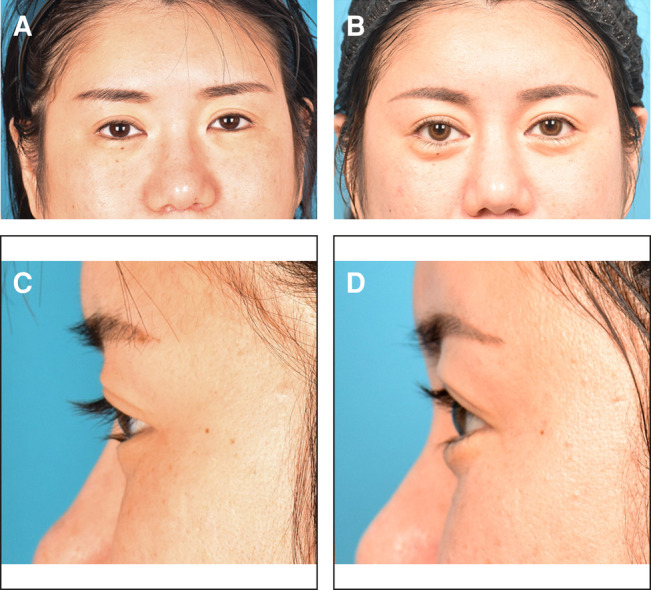
A 36-year-old female requested pretarsal roll fullness of the lower eyelids. (A, C) Preoperative view. (B, D) Postoperative view observed at 2 years and 1 month after pretarsal augmentation of the lower eyelids using fat grafts. She was highly satisfied because the appearance of her eyes became larger than that of her preoperative eyes.

**Figure 4.**
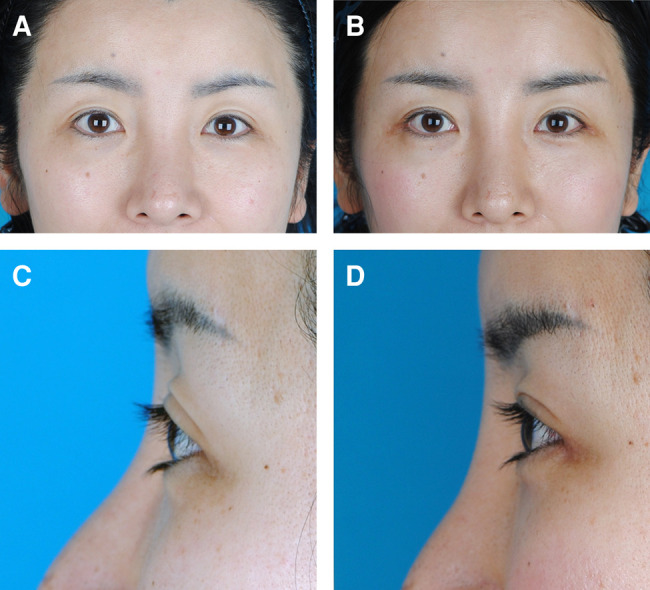
A 39-year-old female requested pretarsal roll fullness of the lower eyelids. (A, C) Preoperative view. (B, D) Postoperative view observed at 1 year and 6 months after pretarsal augmentation of the lower eyelids using fat grafts. She was satisfied because she wanted a small pretarsal roll fullness.

The mean follow-up period was 22.5 months (range, 7-52 months). Ten patients were female, and 2 were male; the mean age was 34.6 years (range, 22-57 years). Pretarsal roll fullness of the lower eyelids increased postoperatively and was maintained in all patients for >6 months. The surgery resulted in enlarged eyes with the newly created pretarsal groove and pretarsal roll fullness, which has an aesthetic effect similar to that of the double eyelid fold in Asian people. Representative clinical cases are shown in Figures 3 and 4.

Changes in the morphometric measurements are shown in Table. The mean projection (1.6 ± 1.0 vs 0.5 ± 0.6 mm), width (7.4 ± 1.5 vs 4.4 ± 3.6 mm), and area (143.5 ± 42.2 vs 32.8 ± 39.6 mm^2^) of the pretarsal roll fullness of the lower eyelids were significantly greater postoperatively (*P* < .05), validating the augmentation efficacy of this technique.

**Table.** Morphometric Data on Pretarsal Roll Fullness

Pretarsal roll fullness (*n* = 12 cases, 24 lower eyelids)	Presurgery Mean ± SD (range)	Postsurgery Mean ± SD (range)	*P*-value
Projection (mm)	0.5 ± 0.6 (0-1.8)	1.6 ± 1.0 (0.8-6)	<.001^a^
Width (mm)	4.4 ± 3.6 (0-10.4)	7.4 ± 1.5 (4.8-11.3)	<.001^a^
Area (mm^2^)	32.8 ± 39.6 (0-110.6)	143.5 ± 42.2 (47.9-222.2)	<.001^a^

The Wilcoxon signed-rank test was used to analyze significant differences between preoperative and postoperative measurements. ^a^Statistically significant.

**Figure 5.**
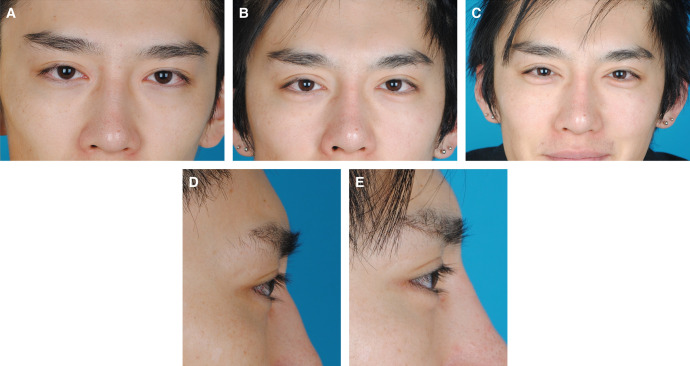
A 27-year-old male requested pretarsal roll fullness of the lower eyelids. The patient also underwent augmentation rhinoplasty and upper lid blepharoplasty. (A, D) Preoperative view. (C) Smile view. (B, E) Postoperative view observed at 10 months after pretarsal augmentation of the lower eyelids using fat grafts. The pretarsal roll fullness of the lower eyelids grew larger compared with the postoperative view observed at 10 months.

**Figure 6.**
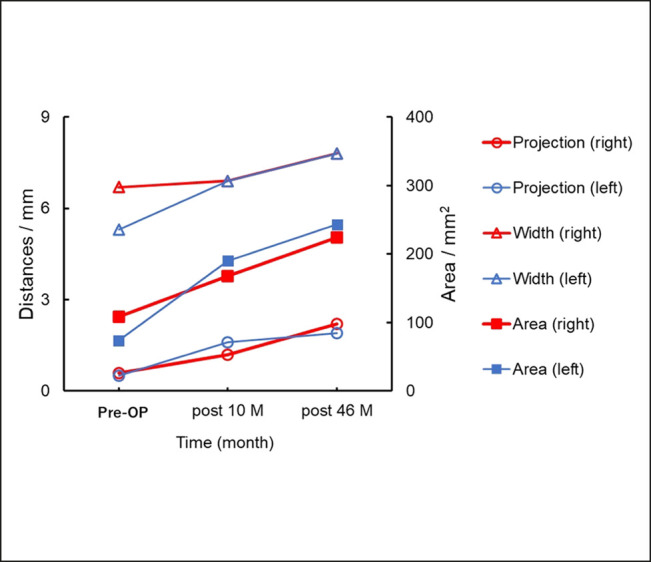
Morphometric changes of overgrowth of pretarsal roll fullness of the case shown in Figure 5. All parameters including the projection, width, and area increased between 10 and 46 months postoperatively, indicating overgrowth of the pretarsal roll fullness of the lower eyelids.

**Figure 7.**
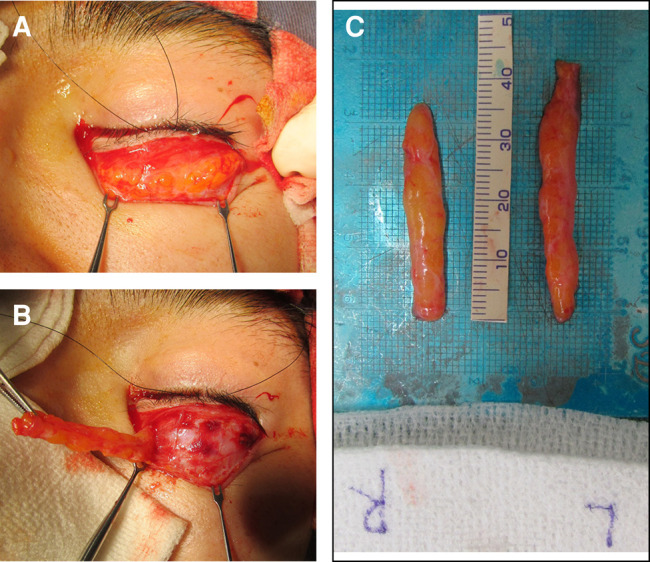
Revision surgery using the excision of implanted fat grafts was performed in the case shown in Figure 5. (A) An implanted fat graft is apparent in the pretarsal region at revision surgery. (B) Excision of implanted fat grafts. (C) The resected specimen (0.49 mL at the right eyelid and 0.52 mL at the left eyelid, respectively) of homogenously distributed implanted fatty tissue.

**Figure 8.**
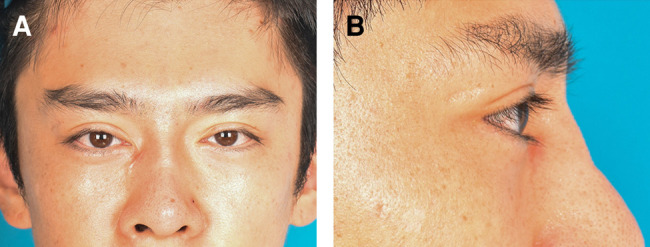
(A, B) The frontal and lateral views of the case shown in Figure 5 observed at 1 year and 3 months after revision surgery.

No early postoperative complications, such as postoperative infection, hematoma formation, graft absorption, graft extrusion, ectropion, hypertrophic scarring, or contour irregularities and granulomas were observed in any of the patients during the 6- to 12-month follow-up period. All patients were satisfied with their aesthetic outcomes. However, overcorrections were observed in 2 cases (16.7%) due to late-onset fat overgrowth (Figures 5, 6), which were corrected by revision surgery using excision or reduction. Macroscopically, the implanted fat was homogeneously located in the subcutaneous tunnel during revision surgery using the subciliary incision approach. Excision or reduction of the implanted fat was easy since the fat tissue was located above the orbicularis oculi muscle (Figure 7). Histologically, the resected fat tissue comprised mature fat cells without atypia. The surgically created pretarsal roll fullness was reduced or eliminated without eyelid deformity after revision surgery (Figure 8).

## DISCUSSION

Currently, several methods can be used to increase pretarsal roll fullness, including hyaluronic acid filler injections,^3^ autologous deep temporal fasciae,^4^ acellular dermal matrices,^5^ implants such as Gore-Tex,^6^ fat injections, tarsal fixations,^7,8^ and muscular overlapping^9^ or muscular suspension.^5^ Our surgical procedure using fat grafts is a simple and reliable alternative for achieving pretarsal roll fullness of the lower eyelids. To our knowledge, this is the first report describing fat grafting for pretarsal augmentation.

Fat grafting is not a new technique and has been previously performed on the eyelid.^10-14^ Indications for fat grafting include a hollow superior sulcus, lower eyelid hollowness, and tear-trough deformity of the lower eyelid. Eyelids provide an excellent vascularized recipient bed, which aids in the survival of free fat grafts. Microfat grafts (2-3 mm in size^12^) or pearl fat grafts (5-6 mm in size,^11^ 4-6 mm in size^14^) are easier to place in a narrow subcutaneous tunnel than lumbrical fat grafts (5-8 mm in diameter and 4 cm long^13^). A previous study showed lower absorption and longer survival of smaller fat grafts than larger fat grafts.^12^ In addition to preventing iatrogenic lacrimal duct injury, the placement of fat grafts in the subcutaneous plane is considered to produce a greater bulging effect on pretarsal roll fullness than their placement in the submuscular plane of the lower eyelid. Despite the superficial placement of these grafts, no unnatural graft visibility was observed in any patient.

Fat grafts have many characteristics that make them ideal fillers for facial soft-tissue defects. Benefits include ease of harvest, minimal donor-site morbidity, and aesthetic benefits such as improved skin quality and natural contour. In contrast, possible complications included unpredictable resorption rates. However, hypertrophy of transplanted fat tissues is less common. Ellenbogen^14^ reported a case in which pearl fat was grafted into the lower eyelid; in the following year, the implanted area became prominent and required resection. Similarly, 2 patients in our study experienced overcorrection due to late-onset fat overgrowth, which was corrected by revision surgery. This phenomenon may be a potential complication of fat grafting.

Fat grafting is a relatively simple technique with few complications; this contrasts with filler or fat injection, which carries the potential risks of arterial embolism, blindness, and cerebral infarction.^3^ Soft-tissue augmentation with a fat graft or fat injection is unpredictable because of varying survival rates. In addition, fat injection is associated with an inherent risk of contour irregularities and granulomas, although the same problems may occur with our technique in long-term follow-up or with a large sample size.

Herein, our technique study showed less absorption and longer survival of fat grafts. With regard to the surgical procedure, precise dissection pockets should be created for fat grafts to achieve smooth contours. The fat grafts were evenly distributed in the closed pockets due to the pressure from surrounding tissues; thus, contour irregularities rarely occurred. In addition, revision surgery was easy to perform after fat grafting using our method because the fat tissue was located above the orbicularis oculi muscle. In contrast, in the case of fat injection, the scope of revision surgery is limited because fat is injected into the orbicularis oculi muscle.

The width of the pretarsal roll fullness was utilized in this study as a measure of size, with 7.4 mm being the mean postoperative width in our study. Based on this, we considered the size to be small if the width was <7.4 mm, and large if it was ≥7.4 mm; however, future studies are needed to determine the ideal pretarsal roll fullness size based on a large sample size. Regardless of the ideal size, our technique provided small or large pretarsal roll fullness at the patient's request. This is beneficial for patients seeking various degrees of pretarsal roll fullness.

Chang and Chang^6^ reported a method for pretarsal augmentation using Gore-Tex implants with excellent outcomes and few complications. However, in our experience, 1 patient who underwent pretarsal augmentation using Gore-Tex implants experienced a foreign body sensation, and the implant was finally replaced with fat grafts.

Surgical methods using orbicularis oculi muscle suspension,^5^ tarsal fixation,^7,8^ and muscular overlapping^9^ have been reported for achieving pretarsal roll fullness. However, the achievable volume enhancement of the pretarsal roll fullness following these procedures is small, and further tissue implantation is required. Our technique does not require orbicularis oculi manipulation and a fat graft provides sufficient volume for achieving the desired pretarsal roll fullness. Additionally, fatty tissues are abundant at suitable donor sites and can be easily harvested.

This study has several limitations. First, the inherent drawbacks of a retrospective design should be acknowledged. Furthermore, definitive conclusions cannot be drawn regarding the specific risk factors for complications because of the relatively small number of patients. Further prospective studies comprising a large number of participants are required to confirm these results; this information would be valuable in understanding that free fat grafts are useful in pretarsal augmentation of the lower eyelids.

## CONCLUSIONS

Pretarsal augmentation of the lower eyelids using fat grafts is a new and effective option, and in this study, it yielded satisfactory results in Asian patients who desired increased pretarsal roll fullness. However, late-onset fat overgrowth after fat grafting into the pretarsal region of the lower eyelid should be taken into consideration.

## Supplementary Material

ojad108_Supplementary_Data
